# Agonistic interactions initiated by adult bottlenose dolphins on Antillean manatee calves in the Caribbean Sea

**DOI:** 10.1371/journal.pone.0295739

**Published:** 2024-01-10

**Authors:** Eric A. Ramos, Jamal Galves, Linda Searle, Zoe Walker, Paul Walker, Nataly Castelblanco-Martínez, Brittany Knowles, Caryn Self-Sullivan, Jeremy J. Kiszka

**Affiliations:** 1 The University of Vermont, Burlington, Vermont, United States of America; 2 Fundación Internacional para la Naturaleza y la Sustentabilidad, Chetumal, Quintana Roo, Mexico; 3 Clearwater Marine Aquarium Research Institute, Belize City, Belize; 4 ECOMAR, St. George’s Caye, Belize City, Belize; 5 Wildtracks, La Isla, Sarteneja Village, Corozal, Belize; 6 Consejo Nacional de Ciencia y Tecnología, Universidad de Quintana Roo, Departamento de Ciencias e Ingeniería, Chetumal, Quintana Roo, Mexico; 7 Oceanographic Center, Nova Southeastern University, Dania Beach, Florida, United States of America; 8 Institute of Environment, Department of Biological Sciences, Florida International University, North Miami, FL, United States of America; PLOS ONE, UNITED KINGDOM

## Abstract

The dynamics and drivers of inter-species interactions in the wild are poorly understood, particularly those involving social animal species. Inter-species interactions between cetaceans and sirenians have rarely been documented and investigated. Here, we report 10 cases of interaction initiated by adult bottlenose dolphins (*Tursiops truncatus*) towards Antillean manatee (*Trichechus manatus manatus*). Interactions were documented through behavioral observations in the wild (*n =* 7) and from the examination of orphaned calves (i.e., tooth rake marks on their body; *n* = 4) that entered a rehabilitation facility, one individual both observed interacting with dolphins and found stranded with bite marks. Bottlenose dolphins were observed interacting with orphan manatee calves and with mother-calf pairs, exhibiting agonistic behavior (*n* = 2), affiliative or neutral behaviors (*n* = 1), but the behavioral contexts of these interactions remain unclear in most cases (*n* = 7). Information on stranded individuals was collected from four calves (of 13 examined calves) recovered in poor condition with bottlenose dolphin tooth rakes and bite wounds on their bodies, one of which died. Injury from bite wounds varied in extent and severity, ranging from superficial scratches leaving rake marks to deep lacerations. Our findings suggest the regular occurrence of agonistic behaviors initiated by adult bottlenose dolphins and directed toward manatee calves. However, the drivers of these interactions remain unknown and need to be further investigated.

## Introduction

Small cetaceans display a diversity of interactions with conspecifics and heterospecifics [1–3]. However, these interactions are often not conspicuous and are therefore not recorded frequently enough to draw conclusions about their function and significance. Interspecies interactions can occur over multiple temporal scales, and involve both affiliative, neutral, or agonistic interactions [4–6]. For example, the formation of mixed-species groups among multiple mammal species is known to provide anti-predator, foraging, and social benefits [7, 8]. In contrast, the function of agonistic interactions remains unclear [2, 4, 9–11].

Common bottlenose dolphins (*Tursiops truncatus*) frequently interact with other species, including other marine mammals such as small cetaceans throughout their range. During these interactions, dolphins can display affiliative behaviors [12, 13], long-term associations [14, 15], epimeletic [16], altruistic [17], and agonistic behaviors [18–21], sometimes involving killing [21, 22]. For example, bottlenose dolphins frequently initiate violent interactions on Guiana dolphins (*Sotalia guianensis*) in several parts of their range [4, 11], and attack and kill (without consuming) harbor porpoises (*Phocoena phocoena*) around the British Isles and off the coast of California [21–23].

Agonistic behaviors among small cetaceans, particularly initiated by common bottlenose dolphins [11, 13, 21, 24], often involves the delivery of blows by the flukes and peduncle, ramming with its rostrum causing blunt force trauma, and bites on animal bodies that leave behind external tooth rake marks [25–27]. The detection of tooth rake marks on stranded animals and the measurement of inter-tooth distances has been essential for confirming interactions between bottlenose dolphins and several species of cetaceans, involving harbor porpoises, common dolphins (*Delphinus delphis*), Atlantic spotted dolphins (*Stenella frontalis*), long-finned pilot whales (*Globicephala melas*), Risso’s dolphins (*Grampus griseus*), and striped dolphins (*Stenella coeruleoalba*) in the Mediterranean Sea, the Atlantic and Indian Oceans [18, 21, 28].

The only reported interactions between cetaceans and sirenians were documented between dugongs (*Dugong dugon*) and several dolphin species [29, 30]. In Shark Bay, Western Australia, Anderson [29] reported four cases of interspecific associations between Indo-Pacific bottlenose dolphins (*Tursiops aduncus*) and dugongs, involving repeated close-proximity swimming between each species but no visible interactions. In one case, a dolphin displayed agonistic behaviors and repeatedly harassed a dugong mother-calf pair. Around the Mozambique Channel Island of Mayotte (SW Indian Ocean), group associations (traveling, resting, and milling) between dugongs and Indo-Pacific bottlenose dolphins, spinner dolphins (*Stenella longirostris*), and Indian Ocean humpback dolphins (*Sousa plumbea*) have been reported [30]. Despite overlap in distribution between common bottlenose dolphins, hereafter bottlenose dolphins, and Antillean manatees (*Trichechus manatus manatus*) along the Atlantic and Caribbean coasts of the Americas [31, 32], to our knowledge, interspecific interactions between these species have not been reported.

Here, we report interactions between adult bottlenose dolphins and Antillean manatee (*T*. *m*. *manatus*) calves between 1999 and 2020 in the Caribbean Sea along the coast of Belize. Data on these interactions were collected through primary observation, reports from the public verified with photos and videos, and physical examinations of orphaned manatee calves upon rescue entry to a rehabilitation facility.

## Methods

### Study area

**Fig 1:**
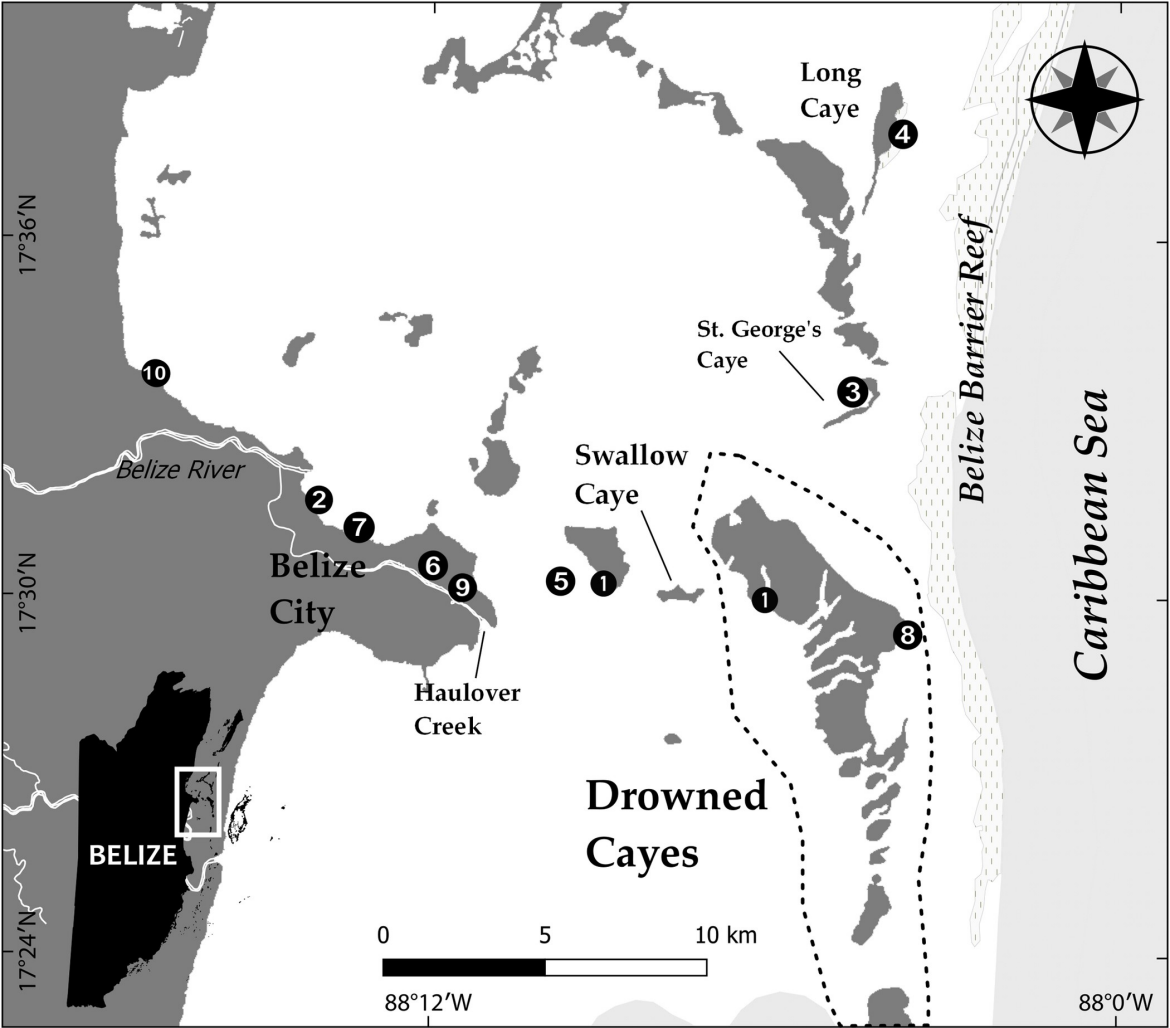
Location of interspecies interactions between adult bottlenose dolphins and Antillean manatee calves (*n* = 10) from 1999 to 2020 in the coastal waters of Belize. Data were visualized with QGIS 3.22.

Data were collected in the coastal waters of Belize City and the Drowned Cayes in Belize (Fig 1). Antillean manatees and bottlenose dolphins are the only marine mammal species regularly found in the shallow coastal habitats along the mainland coast of Belize and within the lagoons of three adjacent offshore atolls [33–35]. All sightings occurred in shallow water habitats (< 5 m depth), including urbanized canal systems on the mainland coast, open areas with silt substrate, areas of dense seagrass beds, and mangrove channels.

### Data collection

To confirm interactions between bottlenose dolphins and manatee calves throughout Belize and explore their significance, we compiled data collected from 1999 to 2020 from various sources providing direct and indirect evidence of dolphin-manatee interactions. All field data were observational and collected by NGOs in Belize under permits granted by the Belize Forestry Department, Belize Fisheries Department, and the Belize Civil Aviation Authority. Manatee calf rescue and rehabilitation work was conducted by Clearwater Marine Aquarium and Wildtracks under a Memorandum of Understanding with the Belize Forestry and Fisheries Department.

#### Observations

Interactions between adult bottlenose dolphins and manatee calves were collected during dedicated boat-based marine mammal surveys from 2001 to 2017 for studies of dolphins and manatees in Belize [36–41]. This included opportunistic observations of at least one member of each species interacting, confirmed by marine mammal experts in the field during dedicated boat-based surveys. Animals were primarily observed from land and from small motorboats (6–8 m long) equipped with outboard engines. For boat-based sightings, observers collected photo-identification photos using DSLR cameras with telephoto lenses (100–400 mm) and sampled the surface behavior of dolphin groups *ad libitum* [42]. Dolphin behavior states were defined as follows based on the literature [43, 44]: *Traveling*: persistent directional swimming; *Socializing*: interaction of two or more individuals of either species within one body length of each other, often involving social displays, chasing, and body contact with other individuals; *Foraging*: repeated diving and/or fast chases in pursuit of prey; and, *Milling*: nondirectional movements. Other behaviors including all affiliative (swimming alongside the calf) and agonistic behaviors (e.g., attempts to submerge the animal, hit it, or launch it into the air) were described where possible.

Two cases were filmed with two small aerial drones: a DJI Phantom 4 Pro (2016) and a DJI Mavic Pro (2020), filming in 4K (3840 × 2160 dpi) at 30 fps. Drones were operated at altitudes of 20 to 150 m by EAR and JG using a remote control while piloting from the shore or from a small boat. The video feed from the onboard camera of the drone was streamed live to an iPad (Apple, Inc.) to monitor animal activity and adjust speed and trajectory of the drone. Observations continued until animals went out of sight, if they moved out of the range of the drone’s flight capacities, and/or until the batteries died. Video footage was stored to a microSD card and was later reviewed in QuickTime Player 7 (Apple Inc.) to describe observed behaviors. In the two cases detected by drone, footage was reviewed to identify behavioral events using continuous all-event sampling [42]. To estimate distances between animals in aerial footage, we used the approximate size of adult bottlenose dolphin as measured from scaled drone footage of an adult coastal bottlenose dolphin (250 cm body length: [45]).

Additionally, videos and images captured by citizen scientists with cell phone cameras were reviewed to identify behaviors in attempts to describe interspecies interactions.

#### Examination of orphaned calves

Evidence of interactions initiated by bottlenose dolphins towards Antillean manatee calves were collected during health assessments of orphaned individuals found by the Belize Marine Mammal Stranding Network (BMMSN). Calf health was evaluated upon intake to Wildtracks in Sarteneja, Belize, the primary rescue and rehabilitation center that raises and releases injured and orphaned manatees. Observations of solitary and potentially orphaned calves were first reported to the BMMSN. In the event a calf was deemed in need of rescue (i.e., alone, small size without mother, visibly injured or ill), it was captured and transported to Wildtracks. Physical capture and handling of manatees for rescue was permitted by the Belize Forest Department and Belize Fisheries Department to JG, ZW, and PW. Upon entry to the rehabilitation center, live calves underwent an initial health assessment and calves that did not survive were necropsied. During examinations, bodies were externally inspected for the presence of anomalous scarring, photographed, and body size was measured with a soft ruler tape according to standard protocols [35].

Calves were animals < 175 cm in total body length [46]. Wounds on the calves were identified as tooth rakes by the presence of clusters of evenly spaced and thin parallel rakes [25]. To confirm their origin, we measured the distances between tooth rakes in photos of manatee wounds and compared these values to previously reported inter-tooth distances of bottlenose dolphins: 5–14 mm [28]; 7–12 mm [18]; and 10.97–12.32 mm [21]. The distances between tooth rakes were measured in *Image J* by tracing a line between two points in adjacent pairs of parallel tooth rakes.

## Results

**Fig 2:**
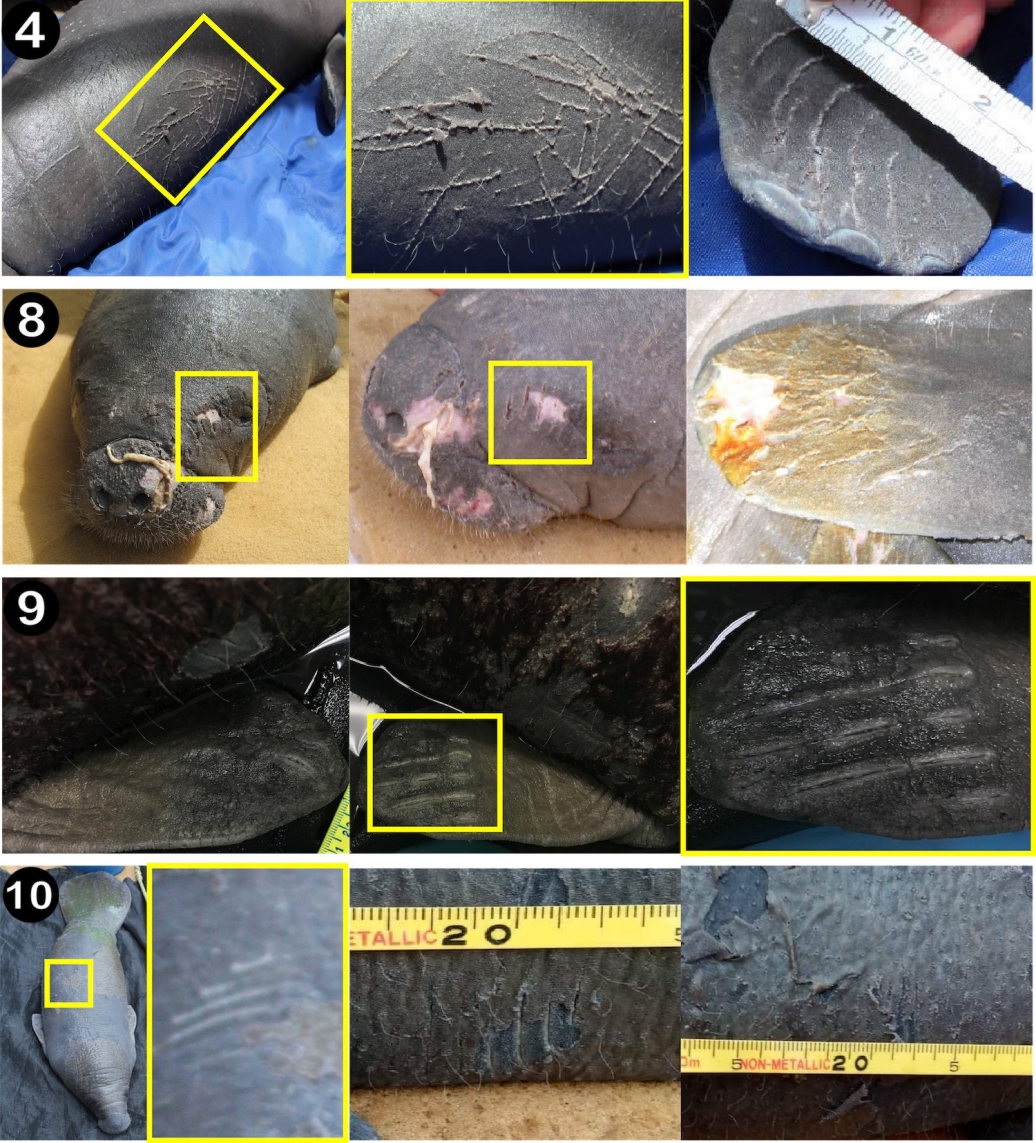
Illustration of wounds and scarring detected on four orphaned Antillean manatee calves rescued in Belize. In Case 4, the calf presented with superficial wounds from tooth rakes, mostly clustered on its right pectoral fin and the right side of its body. The calf in Case 8 sustained major lacerations around her nares and on the left side of her face and presented with numerous tooth rakes on her left pectoral fin. The calf in Case 9 had tooth rakes on its right and left pectoral fins and its body. Tooth rakes were found on the top right and the left side of the calf’s body in Case 10.

**Fig 3:**
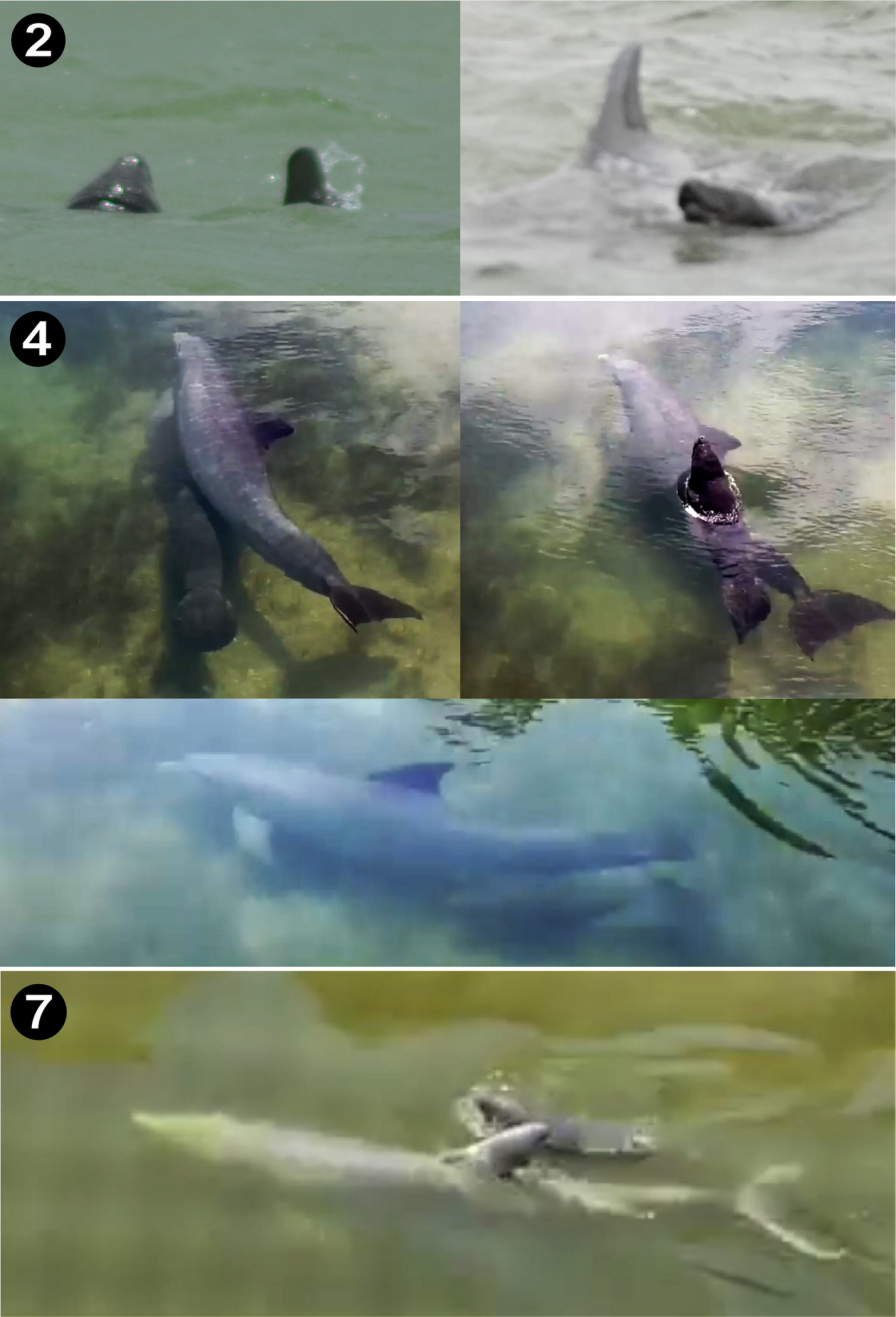
Surface images of bottlenose dolphins swimming with Antillean manatee calves in the shallow coastal waters (< 1.5 m deep) of Belize. In all cases, the single dolphin and manatee calf were seen interacting. The manatee calf was also observed in echelon position. In Case 4, the manatee calf swam underneath the dolphin in echelon position. In Case 7, the calf also swam in echelon position with the dolphin.

**Fig 4:**
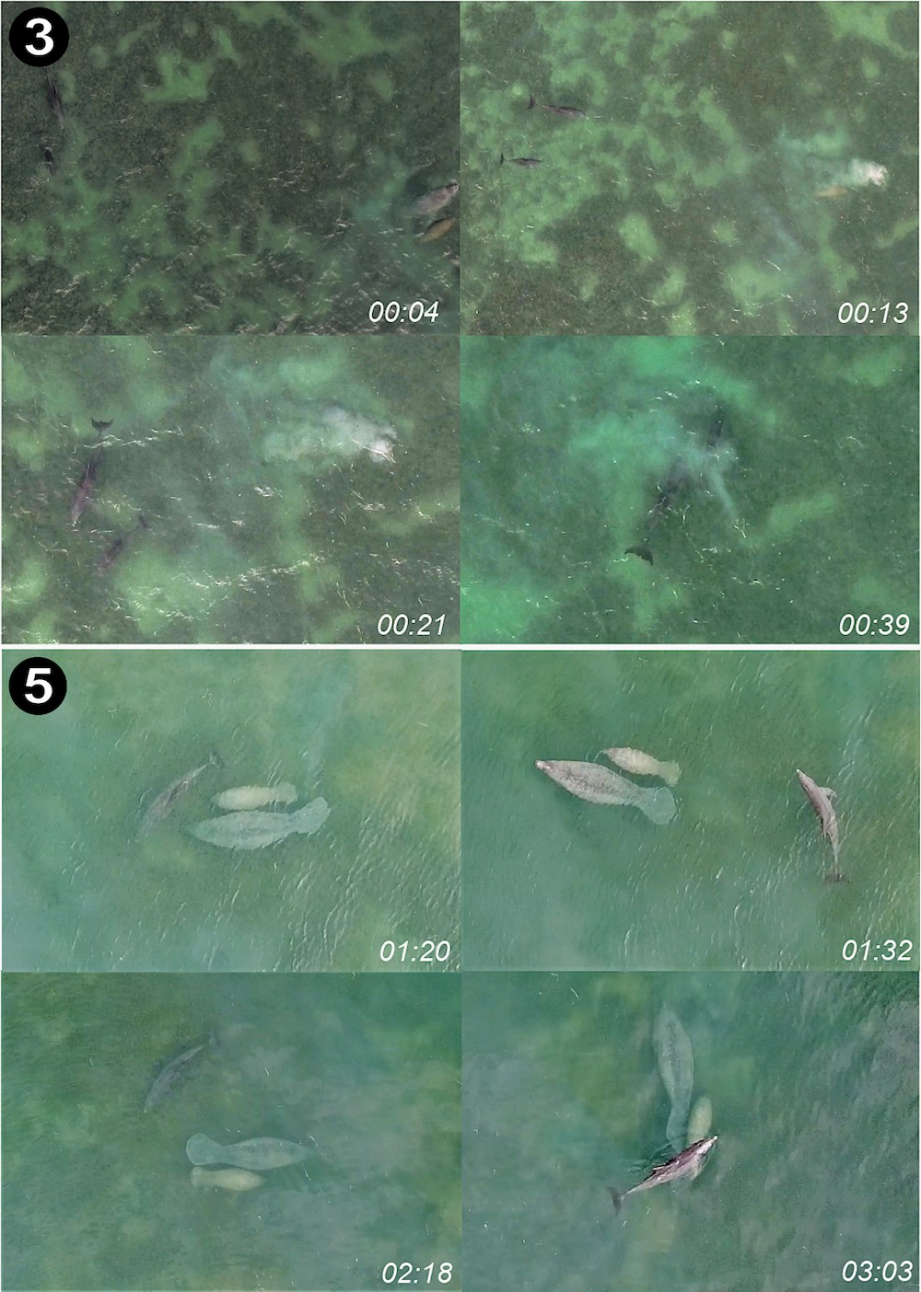
Aerial imagery of bottlenose dolphins interacting with an Antillean manatee mother-calf pair during two flights filmed with small aerial drones. In Case 3, an adult female dolphin and her calf detected a manatee mother-calf in shallow seagrass flats near St. Georges Caye. The dolphins oriented towards the manatees twice before swimming near them briefly and then departing. In Case 5, a single adult dolphin repeatedly encircled a manatee mother-calf pair for at least 4 min near Belize City. Images were exported as screenshots from high-resolution aerial video recordings gathered with a DJI Phantom 4 Pro (Case 3) drone on 30 June 2016 and a DJI Mavic Pro (Case 5) drone on 12 April 2020. Timestamps at the bottom right indicate the time of the exported frame from start of the interaction as captured by the camera of the aerial drone.

We documented 10 cases of interactions between bottlenose dolphins and Antillean manatee calves: 6 cases are based on field observations alone, 3 cases are based on the examination of orphaned manatees (Table 1 and Figs 2–4), and one is based on both field observations and the examination of the manatee calf its interaction with dolphins (Case 4). All calves were found close to the shore (< 10 m): five in canals or creeks on the mainland coast and three near mangrove cayes (Fig 1).

**Table 1:** Details on the 10 cases of interactions between adult bottlenose dolphins and Antillean manatee calves in Belize. F = Female, M = Male, Unk = Unknown, R = Right, L = Left, N/A = Not available. Body lengths with “*” are estimations. Case 4 includes field observations of interactions between an adult bottlenose dolphin and an Antillean manatee calf and the observation of bite marks during health evaluations of the rehabilitation center in Belize, Wildtracks.

Case no.	Date	Detection method	Manatee calf body length (cm)	Sex	Orphan	Location	Habitat type	Outcome
1	9-Aug-1999	Boat	120*	Unk	Yes	Swallow Caye and Drowned Cayes	Mangrove	Unk
2	10-Jun-2015	Boat	130*	Unk	Yes	Driftwood Bay, Belize City	Canal	Unk
3	30-Jun-2016	Drone	>170	Unk	No	St. George’s Caye	Seagrass flats	Unk
4	27-Jun-2018	Shore	108	M	Yes	Long Caye	Mangrove	Rescue/ Death
5	12-Apr-2020	Drone	>160*	Unk	No	West of Belize City	Seagrass flats	Unk
6	5-Jul-2020	Shore	<100*	Unk	Yes	Haulover Creek, Belize City	Creek	Unk
7	19-Jul-2020	Boat	<150*	Unk	Yes	Bella Vista, Belize City	Canal	Unk/ Possible death
8	25-Jun-2009	Boat	118	F	Yes	Heusner Caye	Mangrove	Rescue/ Release
9	15-Aug-2019	Boat	93	F	Yes	Haulover Creek, Belize City	Creek	Rescue/ Death
10	13-Jul-2020	Boat	105	M	Yes	Vista del Mar, Belize City	Canal	Rescue

### Observations: Cases 1–7

#### Case 1

On 9 August 1999, a marine mammal researcher sighted a group of three to five bottlenose dolphins interacting with a young manatee calf from a small motorboat (B. Bilgre, pers. comm., 20 June 2014). The group was first sighted at 11:14 near a channel opening to the Drowned Cayes near the Belize Barrier Reef (Fig 1). The group split into smaller sub-groups, remaining within 30 m of each other for the remainder of the sighting. At 11:41, a manatee calf (probably neonate) was detected and was quickly surrounded by a group of five bottlenose dolphins, including four adults and a juvenile individual. For 15 min, the dolphins exhibited a variety of social and affiliative behaviors directed at the manatee calf including rubbing and supportive behaviors, with the dolphins appearing to push the manatee calf to the surface.

On the same day at 14:00, a single adult bottlenose dolphin and a manatee calf were sighted 5 km east of the previous sighting (Fig 1). The boat followed them as they swam together through a channel between Swallow Caye and Stake Bank Caye and across shallow water seagrass habitats in the Drowned Cayes for 63 min (N. Auil Gomez, pers. comm., 20 June 2014). The dolphin was observed swimming with the manatee calf and displayed affiliative behaviors towards it. The calf first appeared to mill and stayed beneath the peduncle of the dolphin. The dolphin was also seen exposing its ventral surface toward the calf. The observation ended as the pair moved southward into deeper waters at 15:03. Neither animal was re-sighted. Both events most likely involved the same calf based on the short distance between locations and short time between detections.

#### Case 2

On 10 June 2015, an adult bottlenose dolphin was observed from shore by a resident, later assisted by personnel from BMMSN, near a dock immediately south of the Belize River in the Driftwood Bay area in Belize City (Fig 1; R. Codd, pers. comm., 11 July 2014). A manatee calf was observed actively swimming in echelon position alongside the dolphin. The dolphin was observed investigating the calf with its rostrum (Fig 3; See S1 Video). Large splashes and vigorous subsurface movements suggested the dolphin was also harassing the calf. In four short clips totaling 91 sec, dolphin harassment and echelon swimming were observed alternatively.

#### Case 3

At 15:34 on 30 June 2016, a mother-calf bottlenose dolphin pair was observed by EAR interacting with a mother-calf manatee pair in aerial footage captured with a DJI Phantom 4 Pro. The interaction occurred 200 m east of the leeward side of St. George’s Caye (Fig 1; see S2 Video). The calf was large (>170 cm body length), suggesting it was more than a year old. The dolphin pair traveled slowly over seagrass meadows heading northwest as they passed within ∼30 m of a manatee mother-calf pair. As the dolphins passed, the female manatee began to submerge, followed by the calf. The female dolphin and her calf abruptly changed direction swimming directly towards the manatees until within 5–8 m (Fig 4). The dolphin mother-calf pair turned away again and then rapidly approached the manatees. Upon surfacing, both dolphins swam directly towards the manatees, swimming within 1 m of them and possibly making contact. However, water turbidity prevented making further observations. The two manatees remained relatively stationary. After swimming near them, the dolphins turned away and left the area. The entire interaction lasted ∼50 sec, from the moment the dolphins oriented towards the manatees until the dolphins left the area.

#### Case 4

At 10:00 on 27 June 2018, four bottlenose dolphins were observed for approximately 60 min as they interacted with a group of manatees near Long Caye (Fig 1) (D. Smith, pers. comm., 20 July 2018). At the beginning of the sighting, the dolphins swam near a manatee calf and its presumed mother. Several dolphins in the group appeared to harass the mother and attempted to separate the calf from the adult by swimming between the pair and lifting the calf while swimming away. A single adult dolphin continued to swim alongside the calf the manatee calf near the edge of the mangrove caye for several minutes. The calf swam near the dolphin for several minutes, briefly filmed with an Apple iPhone 6 (Fig 3; see S1 Video). The calf swam on the dolphin’s side in echelon position and underneath the peduncle of the dolphin as they both traveled in shallow waters. Several minutes later, a single dolphin was observed displaying aggression and attacking the calf, ramming the calf out of the water into the mangroves and repeatedly biting it. The calf was manually retrieved from the water by JG and transported to the Belize City and then on to Wildtracks for care and rehabilitation.

#### Case 5

On 02 April 2020, a group of manatees (*n* = 11) and a group of foraging bottlenose dolphins (*n* > 4) were filmed by JG using a DJI Mavic Pro on a shallow seagrass bed in the Drowned Cayes (Fig 4; see S2 Video). A mother-calf calf manatee pair entered the area and began feeding, quickly followed by a single dolphin that repeatedly approached the manatee pair. In 4 min and 13 sec of drone video, the dolphin swam in counterclockwise circular paths around the pair 11 times. The two manatees made numerous attempts to depart the area, but the dolphin repeatedly cut off their path (Fig 4). The ability to observe the interaction was interrupted at 30 minutes as the drone had no charged batteries available.

#### Case 6

At ∼16:00 on 5 July 2020, a bottlenose dolphin was seen interacting with a manatee calf in Haulover Creek in Belize City (J. Williams, pers. comm., 6 July 2020). The dolphin repeatedly surfaced near the manatee and appeared to swim over the manatee’s nares when the calf came up to the surface for a breath (Fig 1). The calf repeatedly surfaced swimming alongside the dolphin in echelon position. The interaction lasted for approximately 20 min before both animals were lost from sight.

#### Case 7

At 13:48 on 19 July 2020, a resident of the Bella Vista area sighted a solitary manatee calf (< 150 cm) swimming rapidly behind and on the right side of an adult bottlenose dolphin in a narrow canal (Fig 1; Z. Reich, pers. comm., 21 July 2020). In an 18 sec video of the animals (see S1 Video), the calf remained in close proximity with the dolphin. Extensive searches of the canal and the adjoining canals via boat, drone, and on foot were unsuccessful for resighting both animals. On 24 July 2020, a dead calf was reported in advanced decomposition in a canal approximately 120 m from the location where the dolphin was seen with the calf, but it could not be confirmed if they were the same calf.

### Examination of orphaned manatees: Cases 4, 8–10

Of the 22 manatee calves admitted to Wildtracks from 1999 to 2022, photographs were available for detection of tooth rake marks for 13 animals (2010 to 2020). Four out of these 13 calves (36.4%) presented tooth rakes detected in clusters on different parts of their body (Fig 3 and Table 1), indirectly confirming interactions with adult bottlenose dolphins. Multiple clusters of parallel tooth rake marks (mean = 4.5 clusters per calf; range: 2–8 clusters) found on each calf were sometimes overlapping, indicating multiple bites in the same region. Most bite marks were found throughout the body and on the pectoral fins of calves, with bites on the head in one case. The mean distance between tooth rakes was 8.3 mm and ranged from 5.8 to 11.1 mm (Table 2).

**Table 2:** Information on dolphin tooth rakes found on the bodies of four orphaned Antillean manatee calves recovered for rehabilitation and rescue. The mean distance between tooth rakes is consistent with inter-tooth distances reported for adult bottlenose dolphins: 5–14 mm [28]; 7–12 mm [18]; and 10.97–12.32 mm [21]. The distance between tooth rakes is presented as mean ± standard deviation, range, and the number of measurements taken between adjacent tooth rakes. R = Right; L = Left. Unk = Unknown.

Case no.	Location of tooth rakes	Distance between tooth rakes (mm)	No. of rake clusters
4	Side (R) Pectoral fin (R) Flukes	8.5 ± 1.2 5.8–10.0 *n* = 15	8
8	Body Face Pectoral fin (R)	Unk	6
9	Side (L) Pectoral fin (R/L)	9.2 ± 2.0 6.0–11.1 *n* = 5	2
10	Body	7.0 ± 0.1 6.4–7.8 *n* = 5	2

#### Case 4 (bite mark examination)

The calf (ID: MRP22) observed in the wild in Case 4, entered the rehabilitation facility weighing 20.3 kg with a total body length of 108 cm and was identified as a male. A section of the umbilical cord was still attached to the calf indicating it was a newborn. There were several clusters of tooth rakes on its right side and on the tip of its right pectoral fin, the distal end of its flukes, and oriented both parallel and perpendicular to each other likely representing several separate bites (Fig 2). Some tooth rakes were deep enough to be of concern for causing infection. The calf died 13 days later but it could not be determined if the cause of death was associated with the interaction with dolphins or another cause.

#### Case 8

On 25 June 2009, a manatee calf (ID: MRP08) was observed from shore swimming alone in a depression north of Heusner Caye by the local caretaker of the caye (Fig 1; M. Lambey, pers. comm., 25 June 2009). The calf was observed for several hours to see if it would reunite with its mother or if it would need to be rescued. Several hours later it was still alone, and a rescue operation was organized by BMMSN. A gillnet was used to capture the calf. It was transported by small boat to Belize City and then transferred to Wildtracks.

Upon entry to the rehabilitation facility, the young female calf weighed 25.4 kg with a total body length of 118 cm. Health assessment revealed signs of dehydration and emaciation. Multiple deep skin lacerations were found on the head, right pectoral fin and tail that consisted of clusters of long, thin parallel lines consistent with bottlenose dolphin tooth rakes (Fig 2). The calf fully recovered from these wounds and was raised and rehabilitated at Wildtracks until her successful reintroduction in 2012.

#### Case 9

In the morning of 15 August 2019, a local tour guide and fisherman found a lone manatee calf (ID: MRP24) in Haulover Creek near Belize Mills in Belize City (Fig 1) and reported it to the BMMSN. The animal was exhibiting difficulties to surface and breathe. The 93 cm-long female calf was retrieved but died before it could be transported for rehabilitation. The body was covered in brown algae and presented various circular wounds on the head and throughout the rest of the body. There were a series of tooth rakes at the anterior tip of the right pectoral fin consistent with the bites of an adult bottlenose dolphin (Fig 2). The necropsy revealed the calf was severely emaciated and dehydrated as indicated by the hard and dry fecal matter found in the large intestine. The yellow coloration in the lumen of the stomach was indicative of a lack of recent feeding and suggests the calf had either been unable to eat or was separated from the mother for days. The cause of death is unknown.

#### Case 10

On 13 July 2020, a manatee calf was found swimming alone near Vista del Mar along the coast of Belize City (Fig 1). The calf (105 cm body length) was reported to the BMMSN, and it was successfully located and transported to Wildtracks for rehabilitation. Photographs of the calf (ID: MRP26) taken upon entry to Wildtracks revealed several clusters of lacerations (Fig 2) consistent with bottlenose dolphin tooth rakes. One day following rescue, several additional clusters of bite marks were detected on the abdomen. The calf had two distinct clusters of tooth rakes, one on the tip of each pectoral fin. The manatee recovered and was moved to subsequent stages of release in June 2021.

## Discussion

Inter-species interactions among marine mammal species are common, but their function has rarely been investigated. In this study, we report 10 cases of interspecies interactions initiated by adult bottlenose dolphins on Antillean manatee calves in Belize in the Caribbean Sea. These events were reported using a combination of direct observations of interactions and the examination of orphaned manatees over 21 years, providing support for their regular occurrence. Behaviors previously reported during interspecific interactions of bottlenose dolphins towards other small cetacean species were also observed [6, 11, 20, 27].

The dynamics of these interactions remain poorly understood. For example, it is unclear whether dolphins encountered calves by chance or not, or if they approached and interacted with them out of curiosity, or if they actively sought out lone calves, or if dolphins separated the calves from their mothers. Bottlenose dolphins and Antillean manatees overlap in their range and preferred habitats along the coast of Belize where they forage in similar seagrass habitats [33, 47], which could also increase the likelihood of these interactions. For example, the dolphin mother-calf pair in Case 3 appeared to briefly investigate the manatee mother-calf pair while traveling through the area, suggesting this encounter was by chance. However, regardless of the circumstances by which encounters took place, dolphins clearly initiated interactions with manatee calves in all cases.

Dolphins were observed directing agonistic and aggressive behaviors towards manatee calves in at least 2 cases. For example, in Case 4, several dolphins swam slowly alongside the manatee calf and pushed the calf up to the surface while other dolphins attacked the animal, ramming it with their rostrums and launching the calf into the air. Similarly, in four recovered manatee calves, one or more dolphins had repeatedly bitten the calf. The distances between tooth rakes on the calves measured from scaled imagery fell within the reported range of 5–14 mm for inter-tooth distances of adult common bottlenose dolphins [18, 21, 28], confirming their origin as bottlenose dolphin bites. Several dolphin species display regular intraspecific aggression to conspecific calves, often involving ramming and tossing a calf above the surface in attempts to injure and potentially kill it [5, 19, 48].

In multiple cases (including those with tooth rakes), it was unclear if dolphins were exhibiting agonistic behaviors or if some of these behaviors were affiliative. In Cases 1 and 4, a dolphin attempted to push the manatee calf up to the surface. For Case 1, these behaviors could be interpreted as dolphins engaged in affiliative behavior, and possibly, some degree of alloparental care. Alloparental care, defined as caring for the young of nonrelated conspecific young [49], has been recorded in hundreds of vertebrate species [50, 51], including cetaceans such as sperm whales (*Physeter macrocephalus*) and several delphinid species [52–54]. In contrast, the separation of the calf from its mother in Case 4 did not resemble to alloparental care and suggest that the dolphins may have been trying to separate the calf from the mother. Related and unrelated kin can sometimes attempt to separate conspecific mothers from their calves and have been found with fostered or adopted young through abduction or “kidnapping” of offspring in dolphins [55–57]. Kidnapping behaviors can be detrimental to offspring, sometimes causing death through skull injury, drowning, or emaciation and starvation [57]. Kidnapping is also reported in association with infanticidal behavior involving calf separations from the mother by conspecifics in cetaceans [19, 58]. In Case 2, surface behaviors appearing to be aggressive could possibly represent discipline behaviors such as those displayed by dolphin mothers towards their calves [59]. Additionally, manatee calf proximity to dolphins could be the result of the tendency of marine mammal calves to place themselves alongside their mothers for hydrodynamic benefits or to elicit maternal care [60, 61].

Identifying if interactions with dolphins contributed to the death of manatee calves was not possible. For example, bottlenose dolphins interacting with a manatee calf may unintentionally or intentionally separate it from its mother leading to a mother-calf separation. Additionally, these calves may be physically impaired and have underlying health complications that increase their likelihood of becoming lost or abandoned by their mothers.

Understanding the drivers of interspecies interactions between dolphins and manatees will require further investigation. Future stranded manatees should be systematically examined and complete necropsies should be conducted to identify the cause of death. The use of aerial drones proved highly effective for describing the interaction between dolphins and manatees at a relatively fine spatial and temporal scales [40]. Collecting additional data from photo-identification of individual manatees [62] and drone-based assessments of their body length and condition [19] would contribute to improving our understanding of the occurrence and dynamics of these interactions.

## Conclusions

Our findings reveal the occurrence of previously unreported interspecies interactions between adult bottlenose dolphins and Antillean manatee calves. The behaviors we documented in bottlenose dolphins displayed towards manatee calves are similar to behaviors this species exhibits with calves of their own species and of other species. Understanding the drivers of these interactions is important, but often challenging, particularly for elusive marine mammals occurring in regions where research efforts remain limited. However, further investigating the context and potential drivers of these interactions will be critical to advance our understanding their effects on manatee calf survival in this region.

## Supporting information

S1 Video Compilation of surface video observations of three interactions between bottlenose dolphins and Antillean manatees documented in Belize. (MOV)

S2 Video Aerial drone observations of two different interactions between bottlenose dolphins and Antillean manatee calves. (MOV)
